# BCG-Associated Complications Following Delayed Vaccination in Infants in Saudi Arabia: A Retrospective Cohort Study

**DOI:** 10.3390/vaccines14100878

**Published:** 2026-10-03

**Authors:** Lina I. Alnajjar, Majed Nahari, Nada A. Alsaleh, Reem Binswuidan, Muneerah M. Aleissa, Reham Aljohani, Layan Altalhi, Raheen Almuhlaki, Alhanouf A. Aljaburi, Feryal Alqasimi, Alaa A. Alhubaishi

**Affiliations:** 1 Department of Pharmacy Practice, College of Pharmacy, Princess Nourah bint Abdulrahman University, P.O. Box 84428, Riyadh 11671, Saudi Arabia; lialnajjar@pnu.edu.sa (L.I.A.); mhnahari@kaauh.edu.sa (M.N.); naaalsaleh@pnu.edu.sa (N.A.A.); mmaleissa@pnu.edu.sa (M.M.A.); 2 Pharmaceutical Care Services, King Abdullah Bin Abdulaziz University Hospital, Riyadh 11564, Saudi Arabia; 3 Department of Pharmaceutical Sciences, College of Pharmacy, Princess Nourah bint Abdulrahman University, P.O. Box 84428, Riyadh 11671, Saudi Arabia; rabinsuwaidan@pnu.edu.sa; 4 Pediatric Department, King Fahad Medical City, P.O. Box 59046, Riyadh 11525, Saudi Arabia; rsuliman73@gmail.com; 5 College of Pharmacy, Princess Nourah bint Abdulrahman University, P.O. Box 84428, Riyadh 11671, Saudi Arabia; layanaltalhi2@gmail.com (L.A.); raheen.almutairi@gmail.com (R.A.); alhanoufaljabori@gmail.com (A.A.A.); feryalalqasimi@gmail.com (F.A.)

**Keywords:** BCG vaccine, lymphadenitis, delayed vaccination, BCG-associated complications, Saudi Arabia, immunodeficiency, infants

## Abstract

**Background**: Bacillus Calmette–Guérin (BCG) vaccination is essential for preventing severe tuberculosis in early childhood. The BCG vaccine is administered at birth and carries a risk of disseminated disease, especially in immunocompromised infants. To reduce this risk, Saudi Arabia revised its national immunization policy in 2019, postponing BCG vaccination from birth until six months of age. **Objectives**: This study aimed to evaluate the incidence, clinical characteristics, and management of BCG-associated complications, specifically lymphadenitis, among infants receiving delayed vaccination. **Methods**: This retrospective cohort study was conducted at King Abdullah bin Abdulaziz University Hospital, Riyadh. Electronic medical records of infants vaccinated between 2019 and 2025 were reviewed. **Results**: Data from 994 infants were analyzed. The median age at vaccination was 28 weeks (IQR: 26–30). The overall incidence of BCG-associated complications was remarkably low at 0.5% (n = 5), comprising four cases of lymphadenitis (0.4%) and one case of BCGitis (0.1%). The median time to symptom onset was five weeks post-vaccination for lymphadenitis and 13 weeks for BCGitis. All complications were diagnosed clinically and presented with a mild clinical course. Management was 100% conservative, requiring no antibiotics, surgical intervention, or anti-tuberculosis therapy. All cases achieved complete resolution. **Conclusions**: Delaying BCG vaccination to six months of age significantly enhances safety, resulting in a substantially lower incidence of lymphadenitis compared to historical neonatal administration data. This strategy allows for greater immunological maturity and provides a critical screening window for primary immunodeficiencies, effectively minimizing vaccine-related morbidity while maintaining protective benefit.

## 1. Introduction

The Bacillus Calmette–Guérin (BCG) vaccine is developed from *Mycobacterium bovis* and is the only licensed vaccine for tuberculosis (TB) prevention [1]. An estimated 100 million infants receive the vaccination annually [2]. The vaccination is essential for preventing severe manifestations of tuberculosis, such as tuberculous meningitis and disseminated illness, especially in early childhood [3]. It has been routinely administered in most nations, mostly to newborns through national childhood vaccination programs. Global BCG vaccination policies vary significantly based on tuberculosis (TB) incidence and public health needs. Out of 180 countries assessed, 157 implement universal BCG vaccination for infants.

For example, the UK recommends a single dose at birth, while Brazil supports multiple doses in early childhood. In contrast, 23 countries, including the US and some Western European nations, have either never implemented or have ceased universal BCG vaccination due to declining TB rates [2]. The World Health Organization (WHO) recommends BCG vaccination for neonates and infants in countries or settings with a high incidence of tuberculosis, particularly where the risk of severe childhood TB is substantial [4]. The BCG vaccine in Saudi Arabia was applied as part of the vaccination schedule for all infants and administered at birth [5]. The vaccine has been related to significant adverse effects, including localized ulceration at the injection site, regional lymphadenitis, osteomyelitis, and disseminated disease [5].

Although the BCG vaccine is generally safe, it can cause localized adverse events, primarily BCG-associated lymphadenitis, often manifesting as ipsilateral axillary lymph node enlargement. In severe cases, this can progress to suppurative lymphadenitis, characterized by fluctuation, pus formation, sinus tract development, or caseation. The reported incidence of BCG-associated lymphadenitis varies considerably across populations and vaccination settings. A recent systematic review and meta-analysis of healthy children demonstrated that BCG-associated lymphadenitis remains an uncommon but recognized adverse event following vaccination [6].

Following the introduction of the Danish strain in November 2005, there was a notable increase in BCG-related lymphadenitis prevalence in Saudi Arabia, ranging from 0 to 10.4 cases per 1000 vaccinations [7]. A Saudi prospective observational study also documented BCG-related complications following routine vaccination and highlighted lymphadenitis as an important, localized complication [8]. Molecular confirmation studies from Saudi Arabia have further demonstrated that BCG vaccine strains can be identified in children presenting with vaccine-related adverse events, supporting the association between these clinical presentations and vaccination [9].

A study in Oman, where BCG is administered at birth, involving 88 infants with BCG-related complications found lymphadenitis, with or without abscesses, to be the most common presentation (72.7%), with a median symptom onset of 4 months. Most cases affected the left axilla (76.5%), corresponding to the injection site [10]. Severe disease correlated with early presentation (≤6 months) and immunodeficiency, with a notable proportion (32%) of patients having severe combined immunodeficiency (SCID) or chronic granulomatous disease (CGD), and five deaths reported [10]. Similarly, a study of 171 children in South Korea found axillary lymphadenitis in more than 80% of cases, which typically appeared approximately 2 months after immunization and measured 1.63 cm in size. About 15% of the children experienced recurrent symptoms [11].

Management of BCG lymphadenitis depends on the clinical presentation and severity. Saudi Pediatric Infectious Diseases Society (SPIDS) guidelines provide recommendations for assessment and management, with conservative observation being appropriate for uncomplicated nonsuppurative disease in many cases [12]. Clinical studies have also evaluated needle aspiration as a management strategy in selected cases of BCG lymphadenitis [13].

In 2019, a national policy was introduced in Saudi Arabia recommending postponement of BCG vaccination until 6 months of age to reduce the risk of severe complications, particularly in infants with undiagnosed immunodeficiency disorders such as SCID [3]. Several studies have examined BCG-associated lymphadenitis, but data describing complications among infants vaccinated at six months of age or later in Saudi Arabia remain limited.

Therefore, this study was undertaken to describe the incidence, clinical characteristics, management, and outcomes of documented BCG-associated complications among infants receiving delayed BCG vaccination in a tertiary university hospital in Saudi Arabia.

## 2. Materials and Methods

### 2.1. Study Design

This retrospective cohort study was conducted at King Abdullah bin Abdulaziz University Hospital (KAAUH) in Riyadh, Saudi Arabia, to describe the incidence, clinical characteristics, management, and outcomes of BCG-associated complications among infants who received BCG vaccination at six months of age or older following the implementation of the delayed vaccination policy in Saudi Arabia.

Electronic medical records were retrospectively reviewed to identify infants who received BCG vaccination at KAAUH during the study period from January 2019 to March 2025.

### 2.2. Definitions

Delayed BCG Vaccination: Administration of the Bacillus Calmette–Guérin (BCG) vaccine to infants aged six months or older.

### 2.3. Study Outcomes

The primary outcome was the incidence of BCG-associated complications, including lymphadenitis, among infants who received delayed BCG vaccination. Secondary outcomes were the clinical presentation, time to onset, management, and outcomes of identified complications.

### 2.4. Study Subjects/Participants

Participants in the study were all infants aged six months and older who received the BCG vaccine at King Abdullah bin Abdulaziz University Hospital (KAAUH) and were subsequently assessed for possible vaccine-related lymphadenitis. The study included infants who were six months or older at the time of BCG vaccination. Infants were excluded if they received the BCG vaccine outside KAAUH or under undocumented conditions, or if their medical records lacked sufficient information to confirm diagnosis or treatment outcomes.

### 2.5. Sample Size

The study included all eligible infants who received delayed BCG vaccination during the study period. Given the descriptive nature of the study and the absence of a comparison group, a formal sample size calculation for comparative hypothesis testing was not performed.

### 2.6. Data Collection Procedures and Tool

Patient data were retrospectively collected from electronic medical records and compiled in Excel. Variables included demographics (age, gender, nationality), vaccination details (age/date of BCG administration, injection site), and clinical features (lymph node size/laterality, suppuration, systemic symptoms). Diagnostic data encompassed imaging, microbiology (AFB stain, culture, PCR), and histopathology. Treatment details included medications, surgical procedures, and therapy duration. Outcomes such as resolution, recurrence, complications, and comorbidities (e.g., diabetes, nutritional status) were also recorded.

### 2.7. Data Analysis

Descriptive statistics were used to summarize the study variables. Categorical data are presented as counts and percentages, while continuous variables are expressed as medians and interquartile ranges (IQRs). All analysis was conducted using IBM SPSS Statistics, version 28.0.

## 3. Results

### 3.1. Demographic and Birth Characteristics

Data were collected from a total of 994 infants who received delayed BCG vaccination. The demographic and clinical characteristics are summarized in Table 1. The cohort was nearly balanced in gender, with 515 males (51.8%) and 479 females (48.2%). Most of the participants were Saudi nationals (n = 942, 95%). The median birth weight was 3000 g (IQR: 2700–3300), and the median gestational age was 38 weeks (IQR: 37–40). Prematurity was identified in 11.8% of the cohort (n = 118). Among infants with available delivery-type data (n = 864), 53.7% (n = 464) were delivered vaginally and 46.3% (n = 400) by Cesarean section.

### 3.2. BCG Vaccination Characteristics

The median age at the time of delayed BCG vaccination was 28 weeks (IQR: 26–30). The right arm was the most frequent anatomical site for vaccination (n = 602, 60.6%), while the left arm was used in 38.6% of cases (n = 348). Detailed vaccination characteristics are provided in Table 2.

### 3.3. Incidence of BCG-Associated Complications

The overall incidence of BCG-associated complications in this cohort was 0.5% (5/994). When categorized by specific complication, lymphadenitis occurred in 0.4% of infants (n = 4), and BCGitis occurred in 0.1% of infants (n = 1).

### 3.4. Systemic Clinical Manifestations

Post-vaccination systemic clinical outcomes were rare, as illustrated in Table 3. Systemic symptoms were reported in only three infants (0.3%), with a median onset of 7.25 weeks (IQR: 4–10.5) following vaccination. Respiratory symptoms were noted in a single patient (0.1%) at 8 weeks post-vaccination. No urinary symptoms were reported across the entire cohort.

### 3.5. Characteristics and Management of Complications

The clinical profiles and management strategies for the five infants who developed complications are detailed in Table 4. Among the lymphadenitis cases (n = 4), all presented with axillary node involvement. The median time to onset for lymphadenitis was 5 weeks (IQR: 2–8), whereas the single case of BCGitis presented at 13 weeks post-vaccination. The median node size, reported only for the BCGitis case, was approximately 2 cm. All complications (100%) were diagnosed clinically. Notably, none of the infants with complications had a known history of tuberculosis (TB) exposure. Management was predominantly conservative. All four cases of lymphadenitis (100%) were managed conservatively without the need for antibiotics, aspiration, surgical excision, or anti-TB therapy. The single case of BCGitis also resolved successfully. Overall, a 100% resolution rate was achieved for all reported BCG-associated complications in this study.

## 4. Discussion

The national vaccination policy of Saudi Arabia was changed in 2019 to postpone BCG vaccination from birth to six months of age [3]. This study describes the frequency and clinical course of BCG-associated complications among 994 infants vaccinated under the delayed schedule at a single center. The overall complication rate was 0.5% (5/994), with lymphadenitis occurring in 0.4% and BCGitis in 0.1% of infants. These findings provide an estimate of complication frequency in this delayed-vaccination cohort.

Previous studies from neonatal vaccination settings provide important clinical context for the findings of the present study. Studies from Saudi Arabia and Oman have documented BCG-associated lymphadenitis and other BCG-related complications following vaccination during the neonatal period [7,10]. In the present cohort, BCG vaccination was administered at a median age of 28 weeks, and only five BCG-associated complications were identified among 994 infants. All identified cases had a favorable clinical course and achieved complete resolution. However, these findings should be interpreted as descriptive evidence from a delayed-vaccination cohort rather than as a direct comparison with neonatal vaccination, as the present study did not include a contemporaneous or historical neonatal-vaccination comparison group.

The clinical management observed in this cohort was conservative, with all lymphadenitis cases resolving without antibiotics, aspiration, surgical excision, or anti-tuberculosis therapy. This is consistent with evidence indicating that uncomplicated BCG lymphadenitis can frequently be managed with observation, while intervention may be considered for selected suppurative or persistent cases [12,13]. A multicenter study of BCG vaccine adverse reactions similarly demonstrated that management should be guided by the clinical severity and extent of disease [14].

Disseminated BCG infection is typically related to primary immunodeficiency and is a rare but serious complication. Published reviews emphasize the association between severe BCG disease and underlying primary immunodeficiency disorders [15,16]. There was only one case of BCGitis found in this study. The one case found in this cohort included ipsilateral facial swelling along with localized swelling and drainage at the vaccination site. Interestingly, there were no systemic symptoms in the patient. The condition resolved completely through conservative management, reinforcing the observation that complications in this delayed vaccination group tended to be mild and self-limiting.

The risk of severe BCG disease is particularly important in infants with undiagnosed primary immunodeficiency. Recent studies of disseminated BCG infection have emphasized the potential severity of this complication and its association with underlying immunodeficiency [16,17]. Family history of serious BCG complications may also provide an important clinical clue to an underlying severe primary immunodeficiency [18].

A critical driver for the policy change in Saudi Arabia was the risk of disseminated BCG disease in infants with SCID. Recent data from Riyadh, Saudi Arabia, at King Faisal Specialist Hospital underscored the difference in severe BCG-related complications between the early and delayed vaccination eras. In that study, severe BCG-related complications occurred in 46.1% of patients with SCID during the birth-vaccination era compared with 2.6% during the delayed-vaccination era [3]. Although this evidence supports the rationale for the Saudi policy, it involved a specific high-risk SCID population and should not be directly extrapolated to the general infant population represented in the present study.

Severe disseminated BCG disease may require anti-mycobacterial therapy in affected patients. For example, moxifloxacin has been evaluated as part of the management of disseminated BCGitis in children [19]. However, none of the infants in the present cohort required anti-tuberculosis treatment, which is consistent with the localized and clinically mild nature of the complications observed.

The risk associated with early vaccination is further illustrated by a case involving an infant who received the BCG vaccine before human immunodeficiency virus (HIV) status confirmation. The child was later diagnosed with HIV and developed severe immune reconstitution inflammatory syndrome (IRIS) after starting antiretroviral therapy, leading to suppurative lymphadenitis and tissue breakdown [20].

This study provides data from a general hospital population receiving delayed BCG vaccination. All identified complications resolved, and the lymphadenitis cases were managed conservatively without antibiotics, needle aspiration, or surgery. Strengths include the relatively large cohort and use of electronic medical records. However, the retrospective design may have resulted in missing information; the single-center setting may limit generalizability; some patients may have received care outside KAAUH; and vaccine manufacturer, strain, and batch information was not consistently available.

Most importantly, the absence of an early- or neonatal-vaccination comparison group precluded direct comparison according to vaccination timing and prevents conclusions regarding the impact, superiority, or relative safety of delayed versus neonatal vaccination. In addition, the low number of observed complications limits the ability to draw conclusions regarding predictors of complications. Future studies incorporating a comparable historical or contemporaneous early-vaccination cohort, with standardized case ascertainment and follow-up, are needed to evaluate whether vaccination timing influences the incidence or severity of BCG-associated complications.

## 5. Conclusions

In this retrospective cohort of 994 infants who received BCG vaccination at six months of age or later, BCG-associated complications were uncommon, occurring in 0.5% of infants, with lymphadenitis being the most frequently observed complication. All identified complications had a favorable clinical course, with complete resolution following conservative management and no requirement for anti-tuberculosis therapy, aspiration, or surgical intervention. These findings support a favorable observed safety profile in this delayed-vaccination cohort but do not establish that delayed vaccination is safer than neonatal vaccination because no early-vaccination comparison group was included. Comparative studies are needed to determine whether vaccination at six months or later is associated with a lower incidence or severity of BCG-associated complications than neonatal vaccination.

## Figures and Tables

**Table 1:** Demographic and birth characteristics of infants receiving delayed BCG vaccination (N = 994).

Variable	Category	Total (N = 994)
Gender, n (%)	Female	479 (48.2%)
	Male	515 (51.8%)
Nationality, n (%) *	Saudi	942 (95%)
	Non-Saudi	50 (5%)
Delivery type, n (%) *	C-section	400 (46.3%)
	Vaginal	464 (53.7%)
Gestational Age Category	Prematurity	118 (11.8%)
Birth weight, median (IQR)	3000 (2700–3300)	
Gestational age, median (IQR)	38 (37–40)	

* Data were missing for nationality (n = 2) and delivery type (n = 130). Percentages were calculated based on available data.

**Table 2:** BCG vaccination characteristics among infants receiving delayed BCG vaccination (N = 994).

Variable	Category	Total (N = 994)
Anatomical vaccination site, n (%) *	Left	348 (38.6%)
	Right	602 (60.6%)
Age at BCG vaccination (weeks), median (IQR)	28 (26–30)	

* Data were missing anatomical vaccination site (n = 8). Percentages were calculated based on available data.

**Table 3:** Systemic clinical outcomes following BCG vaccination (N = 994).

Variable	n (%)
Systemic symptoms	3 (0.3%)
Respiratory symptoms	1 (0.1%)
Onset of systemic symptoms (weeks), median IQR	7.25 (4–10.5)
Onset of respiratory symptoms (weeks)	8

No urinary symptoms were reported (0%).

**Table 4:** Clinical characteristics of BCG-related complications (n = 5).

Variable	Lymphadenitis (n = 4)	BCGitis (n = 1)	Total (n = 5)
**Clinical characteristics**			
Gender, n (%)			
Female	2 (50%)	1 (100%)	3 (60%)
Male	2 (50%)	0 (0%)	2 (40%)
Birth weight, median (IQR)	3.38 (3.03–3.73)	3.8	3.46 (3.03–3.89)
Gestational age, median (IQR)	38.75 (37.5–40)	40	38.75 (37.5–40)
Age at vaccination (weeks), median (IQR)	30.25 (26–34.5)	41	33.5 (26–41)
Time to onset (weeks), median (IQR)	5 (2–8)	13	7.25 (4–10.5)
Node location, n (%)	Axillary (100%)	None	Axillary
Node size (cm), median (IQR)		~2 cm (local swelling)	~2 cm
Method of diagnosis, n (%)	Clinical (100%)	Clinical	Clinical
TB exposure, n (%)	0	0	0
**Management**			
Conservative, n (%)	4 (100%)	0	4 (80%)
**Outcomes**			
Resolution, n (%)	4 (100%)	1 (100%)	5 (100%)

## Data Availability

The data that support the findings of this study are available from participating hospitals, but restrictions apply to the availability of these data, which were used under license for the current study, and so are not publicly available. Data are, however, available from the authors upon reasonable request and with permission from the hospitals that participated.

## Abbreviations

AFB	Acid-Fast Bacilli
BCG	Bacillus Calmette–Guérin
CDC	Centers for Disease Control and Prevention
CGD	Chronic Granulomatous Disease
HIV	Human Immunodeficiency Virus
IQR	Interquartile Range
IRIS	Immune Reconstitution Inflammatory Syndrome
KAAUH	King Abdullah bin Abdulaziz University Hospital
KG	Kilogram
PCR	Polymerase Chain Reaction
SCID	Severe Combined Immunodeficiency
SPSS	Statistical Package for the Social Sciences
TB	Tuberculosis
